# Effects of Physical Exercise and Virtual Training on Visual Attention Levels in Children with Autism Spectrum Disorders

**DOI:** 10.3390/brainsci12010041

**Published:** 2021-12-29

**Authors:** Chaoxin Ji, Jun Yang

**Affiliations:** 1 Department of PE, Northeastern University, Shenyang 110819, China; 2 College of Information Science and Engineering, Northeastern University, Shenyang 110819, China; yangjun@mail.neu.edu.cn

**Keywords:** children with ASD, PE, VT, visual attention

## Abstract

This study compared the effects of physical exercise (PE) and virtual training (VT) on the improvement of the visual attention mechanism in children with autism spectrum disorders (ASD). One hundred eighty-nine children with ASD were recruited from Orphan School in Liaoning Province, China. After screening, 100 children ultimately participated in the experiment. Children with ASD were randomly assigned to VT (VT, *n* = 34), PE (PE, *n* = 33) and control group (CG, *n* = 33). The VT group experiment was performed in a virtual environment through the game FIFA21 three times per week for 6 weeks. The PE group played physical football matches three times per week for 6 weeks. Children with ASD in the CG group did not receive VT or PE but only received psychological counseling. Visual attention of children with ASD is evaluated by using the multiple object tracking paradigm (MOT). After 6 weeks of observation, although none of the three groups saw improvements in the correct rate of ring tracking, the observations of the VT and PE groups were significant (*p* < 0.05) compared to the CG group in finding detection rate of probe stimulus. Through MOT tests, VT and PE improved the detection rate of probe stimulus in children with ASD. Therefore, this paper indicates that VT and PE can improve the visual attention ability of children with ASD.

## 1. Introduction

Autism spectrum disorder (ASD) is a neurodevelopmental disorder. At present, cognitive-behavioral therapy (CBT) and pharmacological treatment [1] are the most applied treatment schemes for ASD. However, CBT and pharmacological treatment have certain side effects. Pharmacological resistance can occur as CBT prolongment of patients on the waiting list may worsen symptoms and long-term prognosis. Therefore, the current research mainly focuses on and applies personalized schemes with minimal side effects to treat ASD. Individuals with ASD have obstacles in social and verbal communication and expansibility of thinking, accompanied by typical characteristics of restrictive interests and behaviors [2]. Individuals with ASD generally develop restrictive interests. The restricted interests of autistic individuals are embodied in that they are extremely interested in objects in a narrow range and rigidly organize their behaviors around these interests [3]. When ASD individuals are absorbed in their restricted interests, it is difficult to divert their attention to other objects. Some current intervention studies show that individuals with ASD may have different visual attention patterns for restricted-interest stimulation [4]. Classen suggested that adding pictures of restrictive-interest stimulation to teaching materials in the teaching process can significantly improve the attention level of children with ASD on learning materials, as well as learning effects [5].

As for the visual attention of children with ASD, Richard showed that the visual attention of children with ASD is slower than that of children with typical development [6]. Hochhauser used a change blindness paradigm to evaluate the visual attention mechanism of children with ASD. The main method employed was to train subjects trained by showing them digital real-world images and measure the response time of change. The results showed that the response time of children with ASD was significantly lower than that of children with typical development [7]. Compared to children with typical development, children with ASD performed worse in working memory and IQ tests, but there was no difference in sustained attention between children with ASD and children with typical development in other aspects [8].

Among them, at present, there are many studies using PE to treat children with ASD that have achieved certain therapeutic effects. Tse allowed children with ASD to cycle, and he found that cycling can effectively improve their executive function [9]. At present, more and more people pay attention to the treatment scheme of VT to treat related mental diseases [10]. VT and PE for the treatment of ASD have minimal side effects and can be combined with other methods. VT is a new technology that has emerged within the last 10 years consisting of a set of virtual-reality trainings method established based on a game platform, which is popular with participants because of its entertainment and exercise properties. VT includes many computer-aided training forms. At present, research on VT is mainly focused on the influence of VT on human physiological indexes, such as the research on human strength and balance ability [11]. Saiano studied the street-crossing and street-navigation abilities of patients with ASD through virtual-environment interaction. The research shows that after virtual-environment training, patients with ASD exhibit obvious improvement in street-crossing and street-navigation abilities [12]. Amat designed a training game based on virtual reality, which mainly solved the joint-attention impairment of children with ASD [13]. Mei proposed an attention-training method for children with ASD using virtual-reality games to improve their attention. The research results show that VT can improve the attention of subjects [14]. Virtual-reality games can also effectively improve the communication ability and problem-solving ability of ASD children. Baker used immersive virtual reality as a teaching tool to intervene in social skills and emotional skills of ASD children. He found that immersive virtual reality can effectively improve the sensory preference and visual spatial intensity of subjects [15]. Research by Lecciso showed that VT can effectively improve facial-emotion recognition in ASD children [16]. Abdelmohsen combined social-robot and virtual -reality technology to improve the social skills of ASD children. Research showed that the social capabilities of the subjects was significantly improved [17]. The above results show that VT can effectively improve certain abilities of ASD children.

At present, MOT is often used to discuss the non-target inhibition mechanism in multi-target tracking tasks. For example, in their research [18], Meyerhoff and Papenmeier pointed out the relationship between MOT tasks and visual attention. Although the training effect of VT has been recognized in a general sense and the effectiveness of the training has been proven [19,20], there are still urgent problems to be solved, such as how to reveal the effectiveness of VT at a psychological level and whether the training effect of VT corresponds to the actual training effect at a psychological level. As for the solution to the problem, current evaluation mainly focuses on the sports performance of subjects and infers the effectiveness of VT by observing the sports performance of subjects. However, this cannot reveal the effectiveness of VT and the corresponding reasons for this phenomenon at the psychological level. In order to effectively explore the effect of VT and PE on the visual attention mechanism of children with ASD, this paper uses MOT tasks to explore the changes in visual attention of children with ASD before and after training.

Differences in MOT performance can be attributed to the ability of children with ASD to executive function with limited cognitive resources. Using MOT to test the visual attention mechanism of children with ASD can allow for effective measurement of their information-inhibition ability. The specific research question of this paper is whether VT and PE training can improve the visual attention of ASD children. In this study, we assume that VT and PE have the same training effect; that is, both can improve the visual attention level of children with ASD.

## 2. Materials and Methods

### 2.1. Research Design

In this study, we mainly investigate the non-target inhibition mechanism in MOT and adopt a hybrid research method. In this design, the quantitative data of the test are collected and sorted out, and then the results of quantitative analysis are explained in depth. In quantitative research, the experimental design of repeated VT, PE and CG experiments was adopted.

#### 2.1.1. Participants

**Figure 1:**
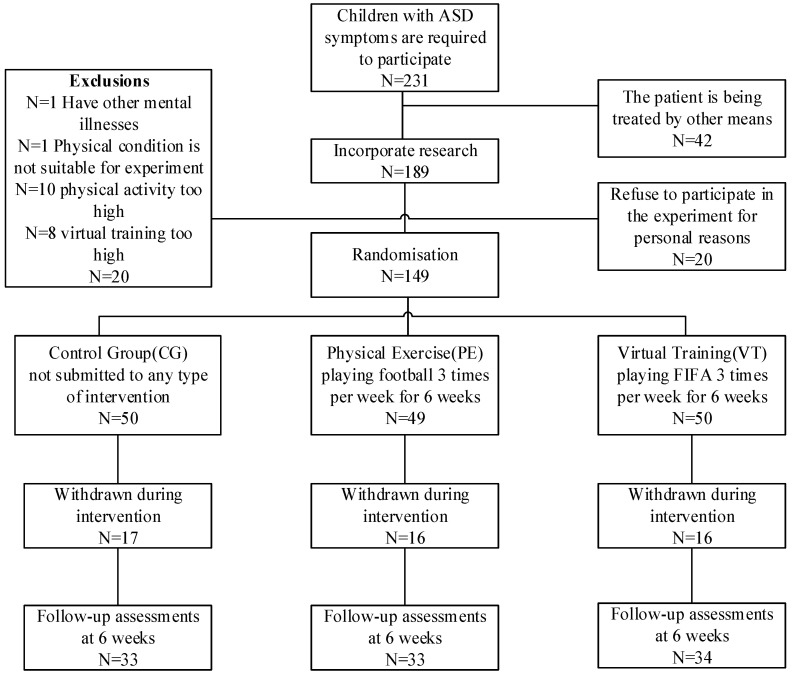
Participant flowchart across the study.

According to the research needs, we visited the Orphan School in Liaoning Province, China, which is a school for children who have lost their parents. In this school, the prevalence of children with ASD is high. Before entering school, children with ASD are diagnosed by mental illness experts at schools in Liaoning Province. All subjects met the current ICD-10 criteria for clinical diagnosis of “pervasive developmental disorders (PDD)” according to the World Health Organization’s (WHO) International Classification of Diseases, 10th edition (ICD-10). All children who met the diagnostic criteria were included in this study. According to the needs of the study, we selected 231 children with ASD to participate in the experiment. Among them, 42 children were excluded because of receiving other treatments, 20 children were excluded because of various conditions, 20 children refused to participate in the experiment for their own reasons and the remaining 149 children were randomly divided into three groups (Figure 1). The average age of the participants was 12.9 years, and most were male (55.0%) (Table 1). Written informed consent was obtained from the guardians of each participant.

Information concerning age and siblings of subjects was recorded. Weight and height were measured, and body mass index (BMI) was calculated for each child. The childhood autism rating scale (CARS) was to evaluate children’s behavior [21]. CARS mainly evaluates the severity of autism symptoms. The scale consists of 15 items, each of which has a score of 1–4, and the total score is the sum of the item scores. If the score is greater than 30, the child is considered to have ASD. A score between 30 and 36 is considered mildly to moderately autistic, and a score greater than 36 is considered severely autistic. The relevant data of the children who participated in the experiment are shown in Table 1. The children with ASD were divided into groups, and experiments were carried out in strict accordance with the experimental requirements, all completed under the supervision of a physical education teacher. VT experiments were also completed under the supervision of a physical education teacher.

#### 2.1.2. Study Methodology

All children with ASD were randomly divided into VT, PE and CG groups. Among them, the intervention method adopted by for the VT group was VT (three times a week, one hour each time). The experiment adopted VR intelligent classroom, with the experiment carried out on with football game FIFA21, an interactive combination of man and machine. The intervention mode of the PE group was football training (three times a week, one hour each session). Children with ASD in the CG group did not receive VT or PE but only received psychological counseling. In this study, no compensation measures were taken. The study was approved by the Ethics Committee of Northeastern University. The study procedure was in accordance with the ethical standards of the institutional and national research committee and with the 1964 Helsinki Declaration and its later amendments or comparable ethical standards.

### 2.2. Production and Presentation of Visual Attention Test Materials

#### 2.2.1. Test Materials

**Figure 2:**
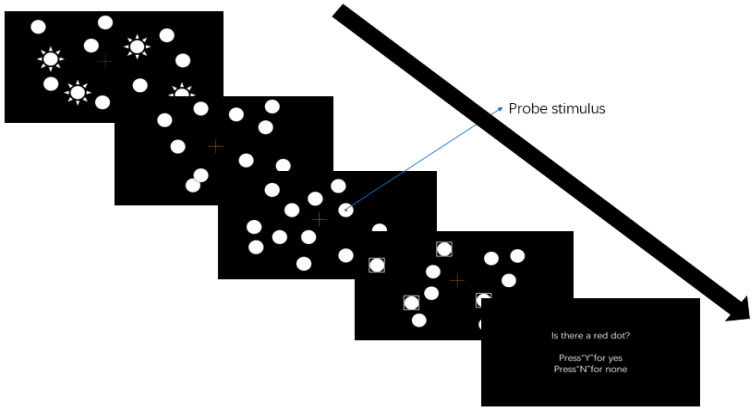
Diagram of the related test procedure.

The test used a computer with black background (screen size: 800 × 600 pixels). The material consisted of 12 white rings (the outer diameter of the ring was 40 pixels, and the inner diameter was 38 pixels), and the probe stimulus was a solid red circle with a diameter of 8 pixels. The relevant test procedures are shown in Figure 2.

#### 2.2.2. Presentation Mode of Test Materials

The test material was presented using an HP N223V computer for which the display was a 17-inch flat CRT display. The MOT program was used in the test, and children with ASD responded with keystrokes through the keyboard of the computer. The recording indexes were the tracking accuracy of the circle and the detection rate of the probe stimulus.

#### 2.2.3. Experimental Design

A mixed design program was adopted in the test.

Intergroup variables: children with ASD (VT, PE, CG);

Intra-group variables: tracking tasks;

Test indexes: correct rate of ring tracking, detection rate of probe stimulus and degree of stimulus. The magnitude is calculated as the detection rate post-test minus the detection rate pre-test.

### 2.3. Test Program

Before the test, participants completed 20 pre-tests to familiarize them with the test process. After the pre-test, the first thing to appear was the test precautions. The test has two missions: tracking the flashing circle and paying attention to whether there is a red dot. After the test starts, 12 white rings appear on the screen, of which 4 white rings will flash three times continuously for 150 ms. Of the remaining 8 rings, 4 regularly swing up and down or left and right (amplitude: 64 pixels), and the others 4 move randomly. After the flicker disappears, all the rings move and can overlap in the process of movement. During the test, the red dot of the probe stimulus appear randomly on the screen. After all the rings stop moving and the selected target is marked, the subjects judge whether they see a red dot. If they see a red dot, they press “Y”; if not, they press “N”. The time of each test is 500 ms, and the test is carried out 160 times in total. After the test has been carried out 80 times, the subjects will have a rest of 2 min. When the rest is over, the computer prompts the subjects to continue the test. In the test, the red dot appears 80 times in total, in a blank area, a moving target area, a moving non-target area and a regular moving non-target area respectively.

### 2.4. Statistical Analysis

SPSS 17.0 was used for statistical analysis, and independent sample *t*-test was used to test the baseline difference of correct rate of ring tracking and the detection rate of probe stimulus pre-test and post-test. The significance level was set at *p* < 0.05 for all analyses. Standard statistical methods were used for the calculation of means and standard deviations. For correct rate of ring tracking, a 3-group (VT/CG/PE) × 2 time (pre/post) mixed ANOVA was performed, with group as the between-subjects factor and time as the within-subjects factor. For detection rate of probe stimulus, a 3-group (VT/CG/PE) × 2 time (pre/post) × 4 stimulus position (blank area/regular moving non-target/, moving target/moving non-target) mixed ANOVA was conducted, with group as the between-subjects factor. Paired-sample *t*-test tested the differences between groups before test intervention.

## 3. Results

### 3.1. Correct Rate of Ring Tracking and Detection Rate of Probe Stimulus

The test data are shown in Table 2. There was no significant difference in the correct rate of ring tracking among different groups: F (1, 97) = 0.339, *p* = 0.562,$\;\eta_{p}^{2}=0.003$. There were significant differences in the detection rate of probe stimulus in different groups: F (1, 97) = 105.71, *p* = 0.001,$\;\eta_{p}^{2}=0.721$. The interaction between test time and group was significant: F (2, 97) = 43.32, *p* = 0.002, $\eta_{p}^{2}=0.530$. In the test, the probe stimulus was divided into four areas, which were blank area, regular moving non-target, moving target and moving non-target. Therefore, the VT group had a higher recognition ability than the PE group when detecting the location of stimulus, which needs to be discussed in the test results.

### 3.2. Correct Rate of Probe Stimulus in Different Positions of Subjects

In the pre-test, an independent sample *t*-test was performed on the location of probe stimulus in VT, PE and CG groups, and it was found that there was no significant difference in probe stimulus in each location among the three groups. Similarly, in the post-test, independent sample *t*-test of the location of the probe stimulus among the three groups showed that there were significant differences between the three groups in moving targets and moving non-targets, as shown in Table 3. In the post-test, the repeated measurement method of analysis of the detection rate of the three groups showed that the main effect of the location of the probe stimulus was significant: F (3, 97) = 305.15, *p* < 0.001, $\eta_{p}^{2}=0.773.$ The interaction between the location of the probe stimulus and group was not significant: F (3, 97) = 1.67, *p* = 0.381, $\eta_{p}^{2}=0.203$. Generally, the detection rates of the three groups were, in the order from high to low: blank area; regular moving non-target; moving target; moving non-target. The post-test showed that the detection rate of moving target and non-moving target in the VT and PE groups was higher than that in CG group, and the difference was significant (*p* < 0.01). The detection rate of the VT group was higher than that of the PE group, but there was no significant difference in any position.

The detection rate of probe stimulus and groups and test time was analyzed by mixed ANOVA. The following results were obtained: (1) The main effect of the location of probe stimulus was significant: F (3, 97) = 203.42, *p* < 0.001, $\eta_{p}^{2}=0.115$. (2) There was no significant interaction between probe stimulus and group: F (3, 97) = 2.05, *p* = 0.231, $\eta_{p}^{2}=0.005$. (3) The interaction between probe stimulus and test time was significant: F (3, 97) = 210.24, *p* < 0.001, $\eta_{p}^{2}=0.227$. (4) The interaction of the three factors was not significant: F (3, 97) = 0.412, *p* = 0.537, $\eta_{p}^{2}=0.005$. In the above tests, we can see that whether it is pre-test or post-test, the detection rate of probe stimulus in different regions varies. The interaction between probe stimulus and groups is not significant, which shows that grouping is not the reason for the difference in detection rate of probe stimulus. The interaction between probe stimulus and test time clearly shows that VT and PE improve the detection rate of probe stimulus.

### 3.3. Probe Stimulus Inhibition Rate in the Subjects

**Figure 3:**
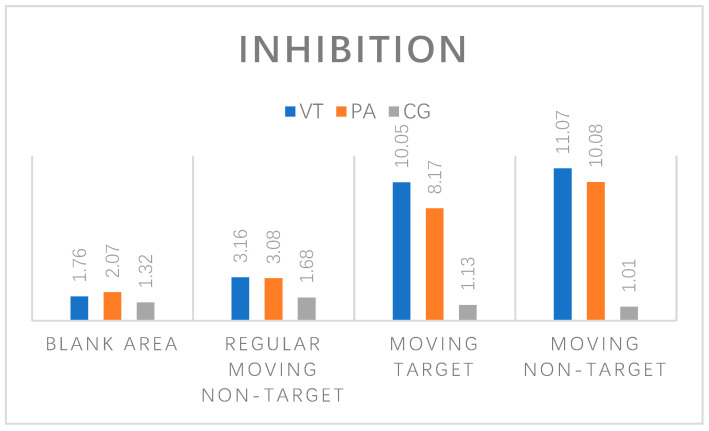
Comparison of the inhibition of different groups in four positions.

In this test, the inhibition of VT group was: blank area, 1.76%; regular moving non-target, 3.16%; moving target, 10.05%; moving non-target, 11.07%. The inhibition of the PE group was: blank area, 2.07%; regular moving non-target, 3.08%; moving target, 8.17%; moving non-target, 10.08%. The inhibition of the CG group was: blank area 1.32%; regular moving non-target, 1.68%; moving target, 1.13%; moving non-target, 1.01%. Pre-test and post-test independent sample *t*-test in the VT group showed that the detection rate of moving target and non-moving target was significantly different (*p* < 0.05), but there was no significant difference in the other two regions. Pre-test and post-test independent sample *t*-test in the PE group showed that the detection rate of moving target and non-moving target was also significantly different (*p* < 0.05). Pre-test and post-test independent sample *t*-test in the CG group showed that there was no significant difference in the detection rate of probe stimulus in the region where detection rate appeared. The relevant comparison results of inhibition are shown in Figure 3.

## 4. Discussion

The research aim of this paper is to investigate whether VT and PE can improve the visual attention level/ability of children with ASD. We hypothesized that after VT and PE training, the visual attention of children with ASD can be improved. Compared with the CG group, after 6 weeks of training, the VT and PE groups showed a higher degree of inhibition for moving targets and non-moving targets. Because the VT group used football games for training, it is necessary for them to effectively distinguish their teammates from other NPC players. At the same time, VT gamers will always maintain inhibition towards other NPC players during the game. Therefore, the inhibition level of VT-trained ASD children was effectively improved. Moreover, the PE group improved correspondingly. Research on the visual attention of children with ASD is attracting more and more attention. Some studies have found that the visual attention ability of children with ASD is worse than that of normal children and that it decreases with an increase in age [22]. In order to eliminate the influence of the time factor, we set up a control group. We found that the detection rate of the probe stimulus in the CG group did not change in the pre-test and post-test. Visual attention did not change; therefore, the time factor was excluded. Some studies have found that the visual attention ability of children with ASD is slower than that of normal children [23]. In this paper, we found that children with ASD can effectively improve the detection rate of probe stimulus, which shows that the visual attention of children with ASD was improved after training. In order to alleviate the symptoms of children with ASD, pharmacological treatment and CBT have been widely used, and the application of PE in the treatment of children with ASD has also been confirmed. However, there are few studies on the application of VT to treat children with ASD. According to the characteristics of children with ASD, this paper designed a mechanism to measure the visual attention of children with ASD by using MOT. The research proves that PE and VT can effectively improve the visual attention of children with ASD. Moreover, a study by Sanger showed that VT has a certain effect on the speed and accuracy of motion decision making. At present, there are many studies using MOT tasks to test the psychological activity level of subjects, such those by Molina [24], Blankenship [25] and Qiu [26], all of which used MOT to monitor subjects’ psychological activity indicators and concluding that MOT can be effectively applied to psychological measurement.

In this paper, we studied the influence of VT and PE on visual attention level and tested visual attention level/ability by MOT, requiring children with ASD to complete MOT pre-test and post-test. Tests showed that VT and PE can effectively improve the visual attention of children with ASD. The research of Alzahabi showed that the limit of subjects’ attention tracking was about 3–5 targets, so the MOT of this test was four targets [27]. Tests show that children with ASD can adapt to the multi-target tracking task, thus proving the effectiveness of the experiment. A prediction model of visual attention of ASD is proposed by Fang based on the prediction method of hierarchical semantic fusion. Through calculation, the proposed model is significantly better than the current significant prediction mode [28]. The results of this study show that VT and PE do not improve the tracking ability of the subjects; that is, compared with the control group, the ability of the two groups to capturing the moving targets was not effectively improved. However, after 6 weeks of VT, the detection rate of subjects was significantly improved, and the detection rate the PE group was also significantly improved. Research shows that during the tracking process in the MOT task, subjects, by identifying different shapes of the target and non-target task, gave an indication of a higher speed of the target shape change than that of the non-target. To that end, the results show that the reason for this is that to track the target, subjects are keen to consciously carry out effective visual attention inhibition on the non-target. At present, there are two viewpoints concerning visual attention inhibition, namely, object-based inference and feature-based inference [29]. Object-based inhibition holds that no matter where the moving non-target moves, visual attention inhibition is always accompanied by the moving non-target and bound with the motion change of the moving non-target. Feature-based visual attention inhibition shows that inhibition is selective, and subjects exhibit an inhibition effect on easily confused non-targets but exhibit no inhibition effect on moving non-targets. Before the pre-test, it was found that the areas with the highest probe stimulus in the three groups were the blank area, regular moving non-target, moving target and moving non-target. This shows that visual attention inhibition is an object-based inhibition; that is, no matter where the moving object goes, the inhibition of visual attention always accompanies the area, except the moving object, and it is easier to detect areas (blank areas) that are obviously different from the target. In the post-test, it was found that there was no change in CG, but the probe stimulus detection rate of moving targets and moving non-targets in the VT and PE groups were significantly improved. The possible reason is that after a period of training, the subjects’ visual attention inhibition changes, which has a stronger inhibition effect on moving targets and moving non-targets; that is, feature-based inhibition probably appears.

## 5. Conclusions

This study compared the effects of VT and PE on visual attention levels in ASD children and tested the visual attention mechanism of ASD children by adding point-detection technology to MOT tasks. The research found that PE and VT do not improve the correct rate of ring tracking but improve the detection rate of probe stimulus. Therefore, PE and VT improve the visual attention of children with ASD at a certain level.

## Figures and Tables

**Table 1:** Demographics of the three groups of children with ASD enrolled in this study.

Characteristic	VT	*n*	PE	*n*	CG	*n*	*p*-Value
	Mean [SD] or *n* (%)		Mean [SD] or *n* (%)		Mean [SD] or *n* (%)		
Age (years)	12.5 [2.36]	34	13.1 [2.97]	33	12.8 [2.69]	33	0.542
F/M	14 (41.18)/20 (58.82)	34	16 (48.48)/17 (51.52)	33	15 (45.45)/18 (54.55)	33	0.368
BMI (kg/m^2^)	21.5 [2.65]	34	22.4 [2.55]	33	20.8 [2.76]	33	0.297
Siblings (none)	28 (82.35)	34	29 (87.88)	33	30 (90.91)	33	0.743
CARS	32.3 [2.98]	34	32.5 [2.65]	33	31.8 [3.32]	33	0.207

Abbreviations: F/M, emale/male; BMI, body mass index; CARS, childhood autism rating scale.

**Table 2:** Correct rate of ring tracking and detection rate of probe stimulus of subjects.

Outcome	Group	Pre-Test	Post-Test	*p*-Value
Correct rate of ring tracking/%	VT	90.56 ± 2.15	90.93 ± 1.59	0.351
	PE	91.02 ± 2.42	90.21 ± 1.97	0.243
	CG	90.14 ± 1.68	90.87 ± 1.93	0.269
Detection rate of probe stimulus/%	VT	42.18 ± 2.09	50.90 ± 3.14	0.030 *
	PE	43.29 ± 1.55	49.43 ± 2.03	0.042 *
	CG	43.17 ± 1.94	42.97 ± 2.04	0.476

Data are presented as mean ± SD, * *p* < 0.05.

**Table 3:** Detection rate of probe stimulus appearing in different positions pre-test and post-test.

Stimulus Positions	Group	Pre-Test (%)	Post-Test (%)	*p*-Value
Blank area	VT	61.49 ± 6.13	63.25 ± 7.05	0.523
	PE	60.03 ± 7.12	62.10 ± 4.25	0.329
	CG	60.79 ± 7.49	62.11 ± 6.14	0.422
Regular moving non-target	VT	43.23 ± 4.32	46.39 ± 5.06	0.201
	PE	44.15 ± 2.67	47.23 ± 4.03	0.103
	CG	46.22 ± 4.75	47.90 ± 3.22	0.307
Moving target	VT	39.14 ± 3.92	49.24 ± 2.58	0.002 *
	PE	38.95 ± 3.04	47.12 ± 3.13	0.005 *
	CG	38.88 ± 4.08	40.01 ± 4.09	0.145
Moving non-target	VT	31.25 ± 6.95	42.32 ± 3.52	0.001 *
	PE	30.28 ± 5.26	40.36 ± 4.19	0.003 *
	CG	31.00 ± 6.12	32.01 ± 5.97	0.325

Data are presented as mean ± SD, * *p <* 0.05.

## Data Availability

Study participant were assured that raw data would remain confidential and would not be shared.
